# The endosome as an effector target to mediate plant immunity?

**DOI:** 10.1093/jxb/erac460

**Published:** 2022-12-19

**Authors:** Aisling Reilly, Angela Feechan

**Affiliations:** School of Agriculture and Food Science, University College Dublin, Belfield, Dublin 4, Ireland; School of Agriculture and Food Science, University College Dublin, Belfield, Dublin 4, Ireland; Institute for Life and Earth Sciences, School of Energy, Geosciences, Infrastructure and Society, Heriot-Watt University, Edinburgh, UK

**Keywords:** Effector, encasement, GEF, hypersensitive response, MVB, powdery mildew

## Abstract

This article comments on:

**Liao W, Nielsen ME, Pedersen C, Xie W, Thordal-Christensen H.** 2023. Barley endosomal MONENSIN SENSITIVITY1 is a target of the powdery mildew effector CSEP0162 and plays a role in plant immunity. Journal of Experimental Botany **74**, 118–129.

**Membrane trafficking is key for different aspects of plant immunity such as the formation of papillae and haustorial encasements. These block ingress or slow the development of filamentous pathogens respectively, while the hypersensitive response (HR) is a localized cell death to halt the spread of such pathogens. Guanine nucleotide exchange factors (GEFs) activate Rab GTPases which regulate membrane trafficking. Now, Liao *et al.* (2023) have found that the GEF Monensin Sensitivity 1 (MON1) is required for components of plant immunity including haustorial encasement formation and the HR. MON1 interacts with the *Blumeria hordei*-secreted effector CSEP0162. Therefore, MON1 is a likely target for the effector CSEP0162 to promote *Bh* infection.**

There are two interconnected layers of plant immunity that protect against pathogen attack. The first is mediated by pattern recognition receptors (PRRs) that initiate PAMP (pathogen-associated molecular pattern)-triggered immunity (PTI). This includes the formation of cell wall appositions (papillae) to prevent entry of filamentous pathogens such as those causing powdery mildew. However, adapted pathogens can overcome PTI by secreting effector proteins (Miller *et al*., 2017). Effector-triggered immunity (ETI) has evolved to counter the susceptibility incurred by fungal effectors directly or indirectly via effector recognition by nucleotide-binding leucine-rich repeat proteins (NLRs) (Jones and Dangl, 2006). This recognition typically leads to a HR characterized by localized programmed cell death (PCD) of the attacked cell (Peterhansel *et al*., 1997).

*Golovinomyces orontii* (*Go*) and *Blumeria hordei* (*Bh*) cause powdery mildew disease on Arabidopsis and barley, respectively (Matsuda and Takamatsu, 2003; Zhang *et al.*, 2012). These fungi directly penetrate the host epidermal cell wall, subsequently developing a feeding structure called a haustorium. Although this feeding structure remains inside the plant epidermal cell, the haustorium is separated from the cytoplasm by a membrane referred to as the extrahaustorial membrane (EHM). It is through this membrane that the transfer of nutrients from the host plant to the fungal pathogen occurs, as well as the delivery of candidate secreted effector proteins (CSEPs) from the fungus to the host (Panstruga and Dodds, 2009; Spanu *et al*., 2010). *Bh* is predicted to encode hundreds of CSEPs produced to hinder plant immunity (Spanu *et al*., 2010; Ahmed *et al.*, 2015). The plant host can block penetration of powdery mildew via papillae formation. However, if penetration is successful, the subsequent haustorium can be ‘cut off’ by encasement of the fungal structure in phenolic compounds and callose or through induction of the HR to prevent nutrient and CSEP transfer (Berkey *et al*., 2017; Rubiato *et al*., 2022).

Membrane trafficking and targeted protein transport are key processes in plant immunity, of which the multivesicular bodies (MVBs)/late endosomes are central (Li *et al*., 2018). For example, secretion occurs from MVBs to form papillae and encasements which was recently found to be dependent on the exocyst subunit EXO70B2 (Ortmannová *et al*., 2022). The delivery of defence proteins and secondary metabolites via extracellular vesicles to the site of pathogen attack at the plasma membrane also occurs from MVBs (Li *et al*., 2018). These components of plant immunity are typical targets for manipulation by pathogen effectors (Gu *et al*., 2017).

## Is endosomal trafficking to encasements blocked by *Bh* effector CSEP0162?

**Fig. 1.**
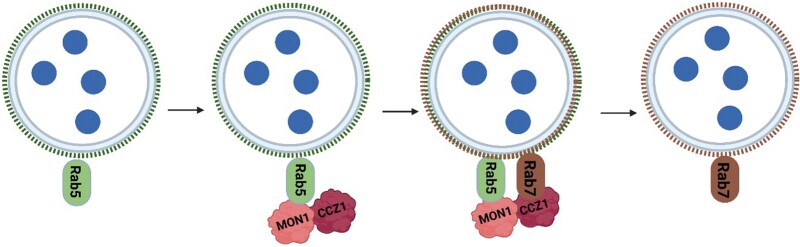
Rab5 to Rab7 transition and endosome maturation mediated by MON1/CCZ1. MON1/CCZ1 is critical for maturation of multivesicular bodies (MVBs). ARA7 is a Rab5 GTPase (green). The MON1/CCZ1 heterodimer (pink/red) acts as a guanine nucleotide exchange factor (GEF) to activate the Rab5 (green) transition to Rab7 (brown) on MVBs. Active Rab7 is subsequently important for the fusion of MVBs to target membranes (after Cui *et al*., 2014). Created with BioRender.com.

**Fig. 2.**
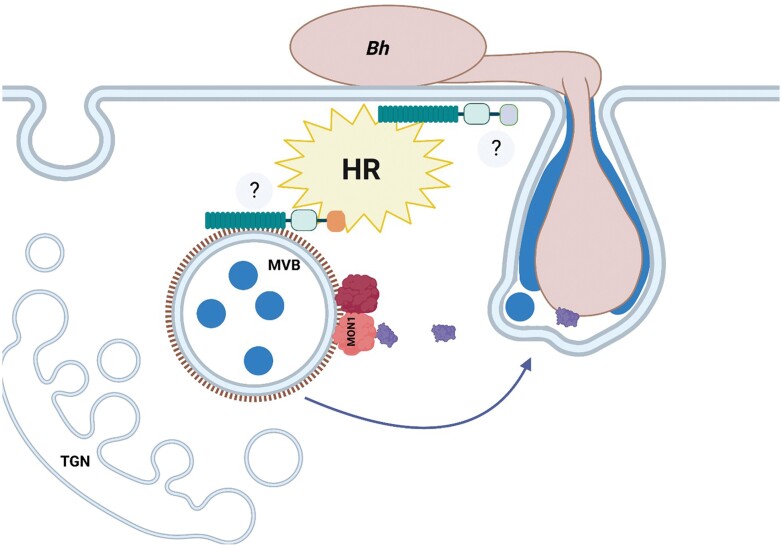
*Bh* effector CSEP0162 targets MON1 which mediates immunity via MVBs/late endosomes. MON1 [together with CCZ (red) as a heterodimer] mediates the maturation and fusion of multivesicular bodies (MVBs). MON1 is required for the formation of haustorial encasements (blue) and cell death/HR (yellow) mediated by CNLs (orange). The HR is activated by TNLs (grey) when MON1 is knocked out. MON1 is a target for the *Blumeria hordei* (*Bh*) effector CSEP1062 (purple) (after Liao *et al*., 2023). Created with BioRender.com.

The knowledge of how membrane trafficking might be targeted by effectors has been furthered recently by Liao *et al.* (2023). The transport, tethering, and docking of target membranes is regulated by Rab GTPase proteins, which interact with membrane protein complexes to specify the tethering of membrane compartments (Gu *et al*., 2017). These Rab GTPases are activated by specific GEFs and are therefore important organizers of membrane compartments, trafficking, and membrane identity (Nielsen *et al*., 2017). The fusion between membranes is facilitated by SNAREs. In Arabidopsis, the secretory syntaxin PEN1/SYP121 (ROR2 is the barley orthologue) is associated with extracellular vesicles and accumulates in both papillae and encasements, whereas the syntaxin SYP122 only accumulates in encasements (Rubiato *et al.*, 2022). Syntaxins form a complex with interacting SNARE proteins to facilitate fusion of membranes and allow the transport of protein cargo to target sites (Nielsen *et al*., 2017). MON1 together with Calcium caffeine Zinc Sensitivity 1 (CCZ1), acts as a GEF for activating Rab7 GTPase (Fig. 1) (Cui *et al*., 2014). Upstream of MON1, VSP9a, which is also a GEF, is required for the activation of ARA7, a Rab5 GTPase. In mammals, Rab5 GTPase is required for the maturation and fusion of MVBs to the plasma membrane to release exosomes (Baietti *et al*., 2012). The MON1/CCZ1 heterodimer mediates the ARA7 Rab5 transition to Rab7, a process critical for MVB maturation (Fig. 1) and vacuolar trafficking (Cui *et al*., 2014). Liao *et al.* (2023) provide evidence that the formation of haustorial encasements is dependent upon MON1 in both barley and Arabidopsis (Fig. 2). It is somewhat unsurprising that a *Bh* effector, such as CSEP0162, targets MON1 in an attempt to block immune responses, since membrane trafficking has a critical role in plant immunity during both pre- and post-invasion defences. However, direct evidence for the role of CSEP0162 in the formation of papillae, encasements and the HR is still required.

## How does MON1 regulate the hypersensitive response?

NLRs can be categorized depending on the N-terminal domain into Toll/interleukin receptor (TIR) domains (TNLs), coiled-coil (CC) domains (CNLs), or Resistance to Powdery Mildew 8 (RPW8)-like CC domains (RNLs). CNLs and TNLs recognize pathogen effectors, whereas RNLs are ‘helpers’ that transduce predominantly TNL signals. CNL resistosomes function as calcium channels at the plasma membrane to induce the HR. It is possible that both CNLs and TNLs ultimately activate calcium channels at the plasma membrane to induce the HR, but the latter is dependent on RNLs (Hu and Zhou, 2022).

What is surprising is that MON1 appears to contribute to the HR (Fig. 2). This was demonstrated by a HR reduction mediated by the CNL *Mla3* (mildew locus a) via knockdown of *HvMON1* in barley. Furthermore, the HR in the Arabidopsis accession No-0 was reduced in *mon1-2* mutants. Liao *et al*. (2023) speculate that this reduced HR response in No-0 is mediated by an unknown CNL. MVBs have previously been implicated to play a role in CNL protein function. In potato, the resistance protein R3a, which normally resides in the cytoplasm, relocates to MVBs when co-expressed with the *Phytophthora infestans* cognate effector Avr3a^KI^. Blocking this localization to the MVB via the vesicle trafficking inhibitors brefeldin A and wortmannin attenuates the R3a-mediated HR (Engelhardt *et al*., 2021). This suggests MVBs act as subcellular compartments from which immune responses can be activated (Li *et al.*, 2018).

Furthermore, the *mon1-1* stunted phenotype in Arabidopsis Col-0 was partially rescued by *eds1-2* which points to a role for TNLs, since Enhanced Disease Susceptibility 1 (EDS1) is required for the HR mediated by TNLs. The loss-of-function mutants of the exocyst subunit *exo70B1* also exhibit autoimmunity, and this requires the truncated TNL, TN2. TN2 is proposed to guard EXO70B1, thus linking MVBs and autoimmunity (Zhao *et al*., 2015).

In a previous study by Ahmed *et al.* (2015), CSEP0162 was found to interact with two small heat shock proteins (sHSPs). Typically, sHSPs assist plant cells to survive stress such as high temperatures by acting as molecular chaperones that facilitate the refolding of misfolded/denatured proteins, and by stabilizing intracellular proteins such as antioxidant enzymes (Schwarczinger *et al.*, 2021). With this in mind, Liao *et al.* (2023) suggest that CSEP0162 may link MON1 to the sHSPs and form an aggresome of the proteins, thereby removing MON1 function. It can also be hypothesized that HSPs are targeted by CSEPs due to their role in the HR response. Schwarczinger *et al.* (2021) found that when barley was pre-exposed to heat stress, they were more susceptible to powdery mildew infection. During high temperatures and elevated reactive oxygen species (ROS) production, sHSPs protect and stabilize antioxidant enzymes that are used to limit ROS production in plant cells. Perhaps fungal effectors such as CSEP0162 ‘hijack’ this stabilizing effect on antioxidant enzymes to reduce ROS during the HR.

## MON1 mediating MVBs and immunity

While the interaction of CSEP0162 with MON1 suggests that this effector targets MVBs to inhibit plant immunity, further study will be required to confirm this role. For example, will silencing CSEP0162 in *Bh* promote both formation of papillae and encasements in barley? Papillae and encasement formation were previously reported to rely on independent pathways, since papillae are labelled with PEN1/SYP121 while encasements are labelled with PEN1/SYP121 and SYP122 (Rubiato *et al.*, 2022). Does MON1 have a role in the formation of both? In addition, will ROS production and the HR be enhanced as a result of silencing CSEP0162? As CSEP0162 may target MON1 to prevent MVB vesicle fusion to the plasma membrane, thereby preventing the HR, it will be interesting to test if the HR mediated by other CNLs (other than *Mla3*) is dependent on MON1. Furthermore, the impact of MON1 on TNL HR induction could be explored since it is possible that HR induction by both NLRs may ultimately be based on MVB fusion and calcium signalling at the plasma membrane. Alternatively, is the link with autoimmunity and TNLs based specifically on those that guard MON1 or a related component of membrane trafficking? Such studies will be important to further elucidate how the MVB links different aspects of plant immunity.
